# Volume imaging to interrogate cancer cell-tumor microenvironment interactions in space and time

**DOI:** 10.3389/fimmu.2023.1176594

**Published:** 2023-05-16

**Authors:** Jorge Almagro, Hendrik A. Messal

**Affiliations:** ^1^ Robin Chemers Neustein Laboratory of Mammalian Cell Biology and Development, The Rockefeller University, New York, NY, United States; ^2^ Division of Molecular Pathology, Oncode Institute, The Netherlands Cancer Institute, Plesmanlaan, Amsterdam, Netherlands

**Keywords:** volume imaging, tissue clearing, intravital imaging, tumor microenvionment, cell-cell interaction, Cell-ECM interaction

## Abstract

Volume imaging visualizes the three-dimensional (3D) complexity of tumors to unravel the dynamic crosstalk between cancer cells and the heterogeneous landscape of the tumor microenvironment (TME). Tissue clearing and intravital microscopy (IVM) constitute rapidly progressing technologies to study the architectural context of such interactions. Tissue clearing enables high-resolution imaging of large samples, allowing for the characterization of entire tumors and even organs and organisms with tumors. With IVM, the dynamic engagement between cancer cells and the TME can be visualized in 3D over time, allowing for acquisition of 4D data. Together, tissue clearing and IVM have been critical in the examination of cancer-TME interactions and have drastically advanced our knowledge in fundamental cancer research and clinical oncology. This review provides an overview of the current technical repertoire of fluorescence volume imaging technologies to study cancer and the TME, and discusses how their recent applications have been utilized to advance our fundamental understanding of tumor architecture, stromal and immune infiltration, vascularization and innervation, and to explore avenues for immunotherapy and optimized chemotherapy delivery.

## 1 Introduction

In the past decade, cancer research and clinical oncology have experienced a paradigm shift driven by the development of immunotherapies to cure patients with aggressive cancers (1). Ever since, cancer research around the world has begun to untangle the diverse functions of the tumor microenvironment (TME), the non-transformed cells and extracellular matrix which constitute the niche in which cancers arise and proliferate.

**Figure 1:**
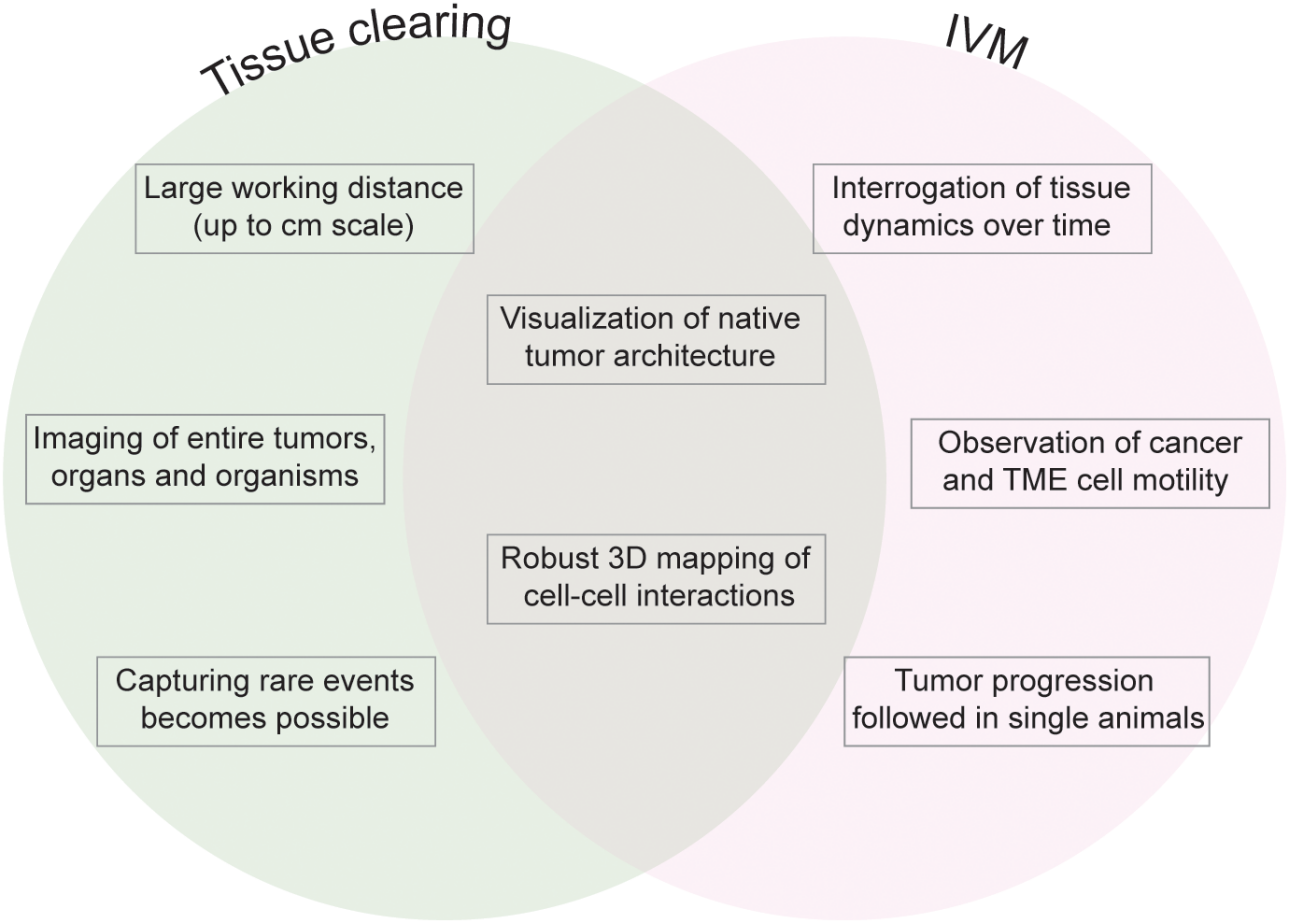
Fluorescence volume imaging in the study of the TME. Schematic outlining the key advantages that are unique to tissue clearing (left), unique to intravital microscopy (IVM, right), and common to both fluorescence volume imaging approaches (center).

Whilst cancer research today appreciates an underlying spatial organization of solid tumors (2), this organization is too complex to be unraveled by a single two-dimensional tissue section, the historic platform for cancer staging, calling for volumetric analyses that capture larger tumor regions if not the entire lesion at cellular resolution (3). This is particularly true for the TME due to the heterogeneity of immune and stromal infiltrates within the complex architecture of solid tumors and the existence of rare components of the TME that despite their sparsity may have important implications in tumor biology. To visualize the full complexity of the TME, high-resolution volume imaging techniques are required (**Figure 1**).

Two powerful technologies for volumetric analyses are *ex vivo* spatial microscopy of tissue-cleared whole organs and intravital microscopy (IVM) of cancer cells in the context of a living organism. Optical clearing to visualize tissues in three dimensions was first developed at the beginning of the 20^th^ century (4) and has dramatically been refined and exploited in the past decade. Originally rediscovered by neurobiology laboratories to achieve 3D maps of the central nervous system, tissue clearing has now been widely applied to study tumor complexity (5). The TME is a dynamic ensemble of cells and molecules that can change dramatically throughout tumor progression (6, 7). By imaging live tissue of animal models, IVM meets the need to scrutinize such dynamics and visualize immune and stromal infiltration and ECM remodeling over time.

This review discusses the recent advances in tissue clearing and IVM to visualize the interaction between cancer cells and cells and molecules in their microenvironment. In addition, we discuss the utilization of these two platforms to investigate the crosstalk between tumor and TME, and to advance our understanding of cancer cell biology in the context of the complex architecture and dynamic organization of cancers.

## 2 Applying tissue clearing to inspect tumors and their TME in 3D

Around seventy tissue clearing techniques have been developed to perform volume fluorescence imaging of tissue resections, whole organs, and entire organisms. This *ex vivo* platform allows for the visualization of cells and macromolecules within the native architecture of their tissue. The first application of clearing in the context of fluorescence microscopy of whole-mount tissue was developed to study fish and *Drosophila* embryos (8) and was later widely applied in neurobiology to study the architecture of the mammalian brain (9). To this day, tissue clearing has been applied to myriad tissues, organs, and organisms including tumors from animal models and human patients (5).

**Figure 2:**
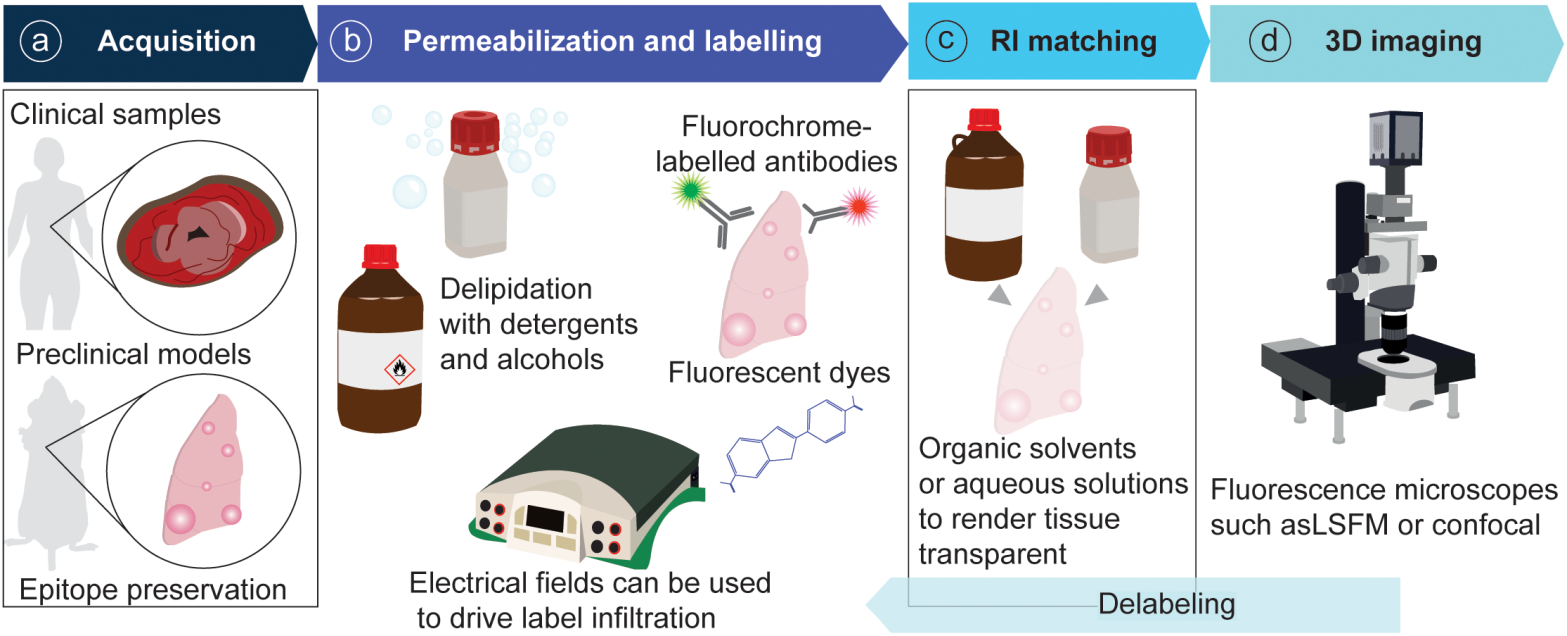
General pipeline of tissue clearing for tumor resections. **(A)** Samples are acquired from patient resections or animal models and fixed to preserve epitopes. **(B)** Samples are permeabilized and labelled with fluorescent probes. **(C)** Samples are rendered transparent with RI-matching solutions. **(D)** Samples are imaged in a fluorescence microscope.

Technically, tissue clearing consists of sample permeabilization and clarification in refractive index matching solutions to render specimens transparent and visualize their entire architecture and internal complexity. The available techniques for tissue permeabilization and refractive index (RI) matching in cancer and other samples have been reviewed extensively (5, 10). Briefly, permeabilization is achieved by lipid elimination using alcohols or detergents, with zwitterionic detergents showing the highest efficacy for delipidation (11–13). Electrophoresis can be used to drive the infiltration of antibodies and dyes into the tissue (14). Clearing is achieved by immersing the sample in an organic solvent or aqueous solution that matches the RI of the tissue. Organic solvents include ethyl cinnamate (ECi), benzyl alcohol/benzyl benzoate (BABB), tetrahydrofuran and dichloromethane (used in DISCO-derived techniques). Aqueous solutions include fructose gradients (as in FRUIT), fructose combined with glycerol and urea (for example, for FUnGI), more complex chemical combinations as in CUBIC (antipyrine, nicotinamide and N-butyldiethanolamine, and other chemicals), as well as commercial solutions like RapiClear® (**Table 1**). The selection of 3D imaging modalities for cleared tissue depends on the sample size and desired optical resolution. Light-sheet fluorescence microscopes provide cellular resolution and can be used to image centimeter-scale samples. Confocal microscopes offer a higher resolution in the xy plane and have been widely utilized to image whole rodent organs and cancer resections. For image rendering and analysis, the free image processing package Fiji can be used (15), and specific software for volume images such as Imaris and Aivia is commercially available (5) (**Figure 2**).

**Table 1:** Technical considerations of tissue clearing techniques.

Approach	Advantages	Disadvantages	Examples
Permeabilization			
Alcohols	Fast delipidation	Often toxic. Photobleaching	iDISCO, 3DISCO, vDISCO
Detergents	Fast delipidation, preservation of endogenous fluorophores	Zwitterionic detergents are preferred, but expensive	FLASH, PACT, CLARITY, SHANEL
Electrophoresis	Homogeneous permeation of labels	Time consuming. Special material required	PACT, CLARITY
Refractive index matching			
Organic solvents	Enhanced transparency	Often toxic. Photobleaching	ECi, BABB, iDISCO, FLASH, FluoClearBABB, vDISCO, TDE
Aqueous solutions	Preservation of fluorochromes	Inefficient clearing of fatty samples	FUnGI, CUBIC, RapiClear, HyClear, HistoDenz, Ce_3_D

Advantages and disadvantages of permeabilization and refractive index procedures used to clear tumors and visualize their microenvironment.

### 2.1 Clearing cancer organotypic cultures

Organoids and organotypic cultures can pose barriers to antibody labeling and microscopic analysis due to their high cellularity and embedded growth in thick culture matrices that prevent access of labeling agents and cause extensive light scattering. Consequently, permeabilization and RI matching have proven useful for complex organoid 3D cultures in which antibody access and light penetration can be limited (11, 16, 17). Tissue clearing has enabled the visualization of glioma and GBM cells in brain organoids using ECi and FRUIT clearing (18, 19). Complex cocultures comprising lung cancer cell organoids with fibroblasts and endothelial cells were cleared with a mixture of polyglycerols, DMSO and urea (HyClear). Each cell line was engineered to express a different, fluorescent protein marker allowing spatial analyses by 3D fluorescent microscopy (20).

### 2.2 Clearing approaches to interrogate the interaction between cancer cells and their epithelial neighbors

Carcinomas originate from epithelial cells and interact and compete with their surrounding healthy epithelium (21). Tissue clearing has been used to study how the epithelial architecture changes upon transformation, and how healthy epithelial cells respond to neighboring tumors. Cytokeratins have been widely used as markers to distinguish epithelia from nonepithelial tissues by 3D imaging. Keratin-8/18 (K8/18) was used to label luminal epithelial cells, and K5 to visualize myoepithelial cells in human prostate biopsies with intraductal carcinoma cleared by iDISCO (22). In prostate cancer samples from FFPE patient biopsies, BABB clearing showed that the K5-expressing myoepithelial cells co-localize with K8-positive tumor regions with a differentiated ductal architecture (23). Both keratins are also expressed in the epithelium of the mammary ductal tree and were visualized together with genetically encoded fluorescent proteins using FUnGI (24). Similarly, fructose glycerol clearing was shown to be applicable to mammary glands with transplanted organoids (25).

In the *MMTV-PyMT* breast cancer model, K8 staining was used to detect spontaneous metastases in lungs cleared by FLASH and to demonstrate their interaction with the airway and alveolar epithelia labelled with podoplanin and surfactant protein C (SFTPC) (11). K7 is a marker of luminal and glandular epithelia in the endometrium, and has been used together with CUBIC clearing to investigate the architecture of occluded glands and adenomyosis in humans, associated to cervical cancer (26). Genetically KrasG12D transformed cells in the pancreas, liver and lung were visualized through a combination of K19 immunolabelling with antibody staining for red and green fluorescent protein (RFP, GFP) lineage tracers (27) (**Table 1**).

### 2.3 Tissue clearing techniques for brain cancer models

Many clearing techniques were established to clear the brain and study its anatomy in pathophysiology (9). Hence, the interaction of different brain tumors with cells of the normal brain parenchyma, including neurons and glia, could be readily investigated in 3D.

The astrocyte marker glial fibrillary acidic protein (GFAP) has been used to visualize the interaction of glial cells with GBM models in mouse brains cleared in tetrahydrofuran, urea, azide and nycodenz (28), and with CUBIC and iDISCO (29). Co-invasion of human glioma cell lines and astrocytes has been investigated in brain slices labelled with anti-GFAP antibody and imaged after clearing in α-thioglycerol, fructose (30) and RapiClear (31, 32). These studies also labelled laminin to image the interaction between tumor cells and the surrounding extracellular matrix, and vimentin, which marks processes of glioma cells reaching into the surrounding environment (31, 32). Astrocyte interaction with breast cancer tumor metastases in the brain administered by intracarotid or intracranial injection was visualized with CUBIC and PACT clearing (33). The interaction between tumors and axons in 3D has been achieved through lipid staining and clearing with urea and triton (34), and staining for myelin basic protein (MBP) in GBM cleared with CUBIC and iDISCO (29) (**Table 1**).

### 2.4 Clearing the tumor immune microenvironment

Tissue clearing constitutes a useful approach to accurately assess the infiltration of immune cells into the tumor. A general visualization of immune infiltration can be achieved with the pan-immune cell marker CD45, as demonstrated in a mouse glioblastoma (GBM) model cleared with iDISCO and CUBIC (29). The infiltration of fluorescently labelled tumor-associated macrophages (TAMs) has been visualized in murine lung cancer models cleared with CUBIC (35). Macrophage infiltration in mouse lung adenocarcinoma tumors generated by intravenous injection of A549 cells was also achieved using an Iba-1 antibody and CUBIC clearing (36). Primary and secondary tumors from anaplastic thyroid tumors achieved by orthotopic and intravenous injection into mice respectively were cleared with CUBIC, and TAMs visualized using a Macrin-VT680XL nanoparticle (37) (**Table 2**).

**Table 2:** Markers to label the TME of cleared tumors.

Antibody	RRID/clone	Clearing	Target	Tumor
α-SMA	AB_476977	CUBIC	Perivascular cells	A549 orthotopic lung tumors (36), OS-RC-1 brain metastases in whole cleared mice (41)
α-SMA	AB_476746	Fructose	Perivascular cells	MMTV-Her2/Neu mammary tumor (38)
CD3	Clone LN10	CLARITY	T cells	Breast cancer (39)
CD3	AB_389323	ECi	T cells	HNSCC (40)
CD8	Clone 2.43	Fructose	T cells	Mammary tumor (38)
CD31	AB_726362	Glycerol, TDE	Blood endothelium	Orthotopic glioblastoma (42) Melanoma xenografts (43)
CD31	AB_312909	Fructose	Blood vessel endothelium	MMTV-Her2/Neu and 4T1 mammary tumors (38)
CD31	AB_61339	FocusClear	Blood vessel endothelium	Human colon adenocarcinoma biopsies (14)
CD34	Clone QBEnd/10	FocusClear	Blood vessel endothelium	Human colon adenocarcinoma biopsies (14)
CD42c	Cat. no. X649 Emfret Analytics	CUBIC	Platelets	A549 orthotopic lung tumors (36)
CD66b	AB_2893284	ECi	Neutrophils	Human HNSCC (40)
Factor VIII	Agilent	CUBIC, BABB, TDE	Coagulation cascade	Melanoma xenografts (43)
GFAP	AB_10013382	THF+nycodenz, CUBIC, iDISCO	Astrocytes	U251-MG orthotopic GBM (28, 29)
Iba-1	AB_2687911	CUBIC	Macrophages	Lung adenocarcinoma (A549 IV) (36)
K5	AB_869890	iDISCO	Myoepithelial cells	Prostate DCIS (22)
K7	Clone EPR1619Y	CUBIC	Luminal and glandular epithelia	Uterine adenomyosis (26)
K8/18	AB_141750	iDISCO	Luminal cells	Prostate DCIS (22)
K8	AB_531826	FLASH	Tumor cells, airways, alveolar cells	Lungs with MMTV-PyMT metastases (23)
K19	AB_2133570	FLASH	Ductal epithelia	Pancreas, lung and liver biliary tree with transformed cells (27)
Lyve-1	AB_881387	DCM DBE	Lymphatic vessels	Human bladder cancer FFPE (44)
Lyve-1	AB_301509	RapiClear 1.52	Lymphatic vessels	Mouse and human PanIN and PDAC (45)
MBP	AB_956157	CUBIC, iDISCO	Myelin	GBM (29)
MECA-79	AB_395099	Dent bleach and BABB	High endothelial venules	Fibrosarcoma and 4T1 xenografts (46)
Podoplanin	AB_2565183	RapiClear 1.52	Lymphatic vessels	Mouse and human PanIN and PDAC (45)
Podoplanin	AB_2268062	FLASH	Alveolar cells	Lungs with MMTV-PyMT metastases (11)
SFTPC	AB_1857425	FLASH	Alveolar type I cells	Lungs with MMTV-PyMT metastases (11)
VEGFR	AB_2167245	CUBIC	Angiogenesis	A549 orthotopic lung tumors (36)
Vimentin	AB_11212377	RapiClear	Glioma cell processes	Glioma invasion assay in brain slices *in vitro (* 31, 32 *)*

α-SMA, alpha-smooth muscle actin; CUBIC, clear, unobstructed brain/body imaging cocktails and computational analysis; CD, cluster of differentiation; MMTV, murine mammary tumor virus; CLARITY, clear lipid-exchanged acrylamide-hybridized rigid imaging / immunostaining / in situ-hybridization-compatible tissue hydrogel; ECi, ethyl cynammate; HNSCC, head and neck squamous cell carcinoma; TDE, thiodiethanol; BABB, benzyl alcohol/benzyl benzoate; THF, tetrahydrofuran; iDISCO, immunolabeling-enabled imaging of solvent-cleared organs; GFAP, glial fibrillary acidic protein; GBM, glioblastoma; Iba-1, allograft inflammatory factor 1; IV, intravenous; K, cytokeratin; DCIS, ductal carcinoma in situ; FLASH, fast light-microscopic analysis of antibody-stained whole organs; PyMT, polyomavirus middle-T; Lyve-1, link domain-containing hyaladherin; DCM, dichloromethane; DBE, dibenzyl ether; FFPE, formalin-fixed paraffin-embedded; PanIN, pancreatic intraepithelial neoplasia; PDAC, pancreatic ductal adenocarcinoma; MBP, myelin basic protein; MECA-79, peripheral node addressin antibody; SFTPC, surfactant protein C; VEGFR, vascular endothelial growth factor. Clone or catalog number and/or provider were indicated for antibodies for which RRID identifier was not available.

Clearing and imaging of infiltrating T cells has also been achieved. CD8^+^ cells and their contribution within the heterogeneous expression landscape of immune checkpoint molecule PD-L1 was performed in mouse mammary tumor models cleared in fructose (38). CD3 staining was used to evaluate the infiltration of T cells in formalin fixed paraffin-embedded (FFPE) breast cancer patient biopsies cleared with CLARITY (39), and surgical resections of patients with head and neck squamous cell carcinoma (HNSCC) were stained with antibodies against CD3 for T cells and CD66b for neutrophils before clearing with ECi (40) (**Table 2**). Recent work using FLASH has revealed differences in T and B cell infiltration in whole tumour-bearing lungs, and has proved useful in the identification of tumour-adjacent immune structures such as tertiary lymphoid structures which are defined by their histology (47).

The study of other immune cell types in different types of tumors remains to be performed, but specialized techniques such as EMOVI have been used to detect immune cells in non-cancerous samples, including antigen presenting cells using MHC-II antibodies, T cells with a CD3 antibody, and B cells and dendritic cells with antibodies against CD21 and CD35 in lungs, lymph nodes, adipose tissue, kidney, brain, liver and heart (48), giving a promising outlook for the future study of these infiltrates and their interaction with cancer cells.

However, fluorescence imaging of large volumes is usually restricted to 4-5 markers, which does not meet the requirements for an in-depth characterization of immune infiltrates. The development of multiplex imaging technologies compatible with 3D imaging brings promise to the spatial characterization of immune infiltration within the tumor volume (See section 6).

### 2.5 Visualizing the tumor vasculature with tissue clearing

Tissue clearing has been used extensively to map the vasculature of biological samples including tumors. In addition to antibodies that specifically label the endothelium or perivascular cells, vessels can be easily stained in animal models through intravenous injection of fluorescent dextran or lectin.

Lectin dyes bind to the luminal side of endothelial cells and have been applied to image the architecture of the vascular network in mouse models of glioma using 3DISCO (49) and FluoClearBABB (50), HNSCC xenografts from human cell lines using the commercial Binaree clearing kit (51), human hepatoma xenografts (52) and human pancreatic ductal adenocarcinoma (PDAC) xenografts in mice cleared with CUBIC (53), and a genetic PDAC mouse model cleared with FLASH (11). Lectins have also been used to label the vasculature of entire mice with primary and metastatic orthotopic tumor models for breast cancer, PDAC, and lung cancer cleared with vDISCO (48). Lectins can be used in combination with antibody labelling as shown in a mouse model for GBM, in which the vasculature was stained both with lectin and a CD31 antibody and the tumors cleared in 80% glycerol (54). Tumor perfusion and drug penetration throughout the tumor can also be evaluated using lectin labelling. HER-2 overexpressing breast cancer xenografts have been imaged to evaluate the penetration of a fluorescent HER-2-targeting antibody (trastuzumab) in tumors cleared with BABB (55). Similarly, in HeLa subcutaneous xenografts, lectin was used to map the distribution of nanoparticles in tumors cleared with CUBIC (56).

Dextran can also be used to label blood vasculature, as shown in A549 lung tumors cleared with CUBIC-plus (57). Dextran permeates through fenestrated vessels and can hence be used to evaluate the integrity and permeability of the vasculature, as demonstrated in GBM xenografts cleared in ECi (38).

Widely-used epitopes to label vasculature include α-smooth muscle actin (α-SMA, labelling pericytes), used in an orthotopic model of lung cancer cleared with CUBIC (36), in a breast cancer model cleared with fructose (39), and in a renal carcinoma brain metastasis model visualized in whole mice cleared with CUBIC (58). CD31 to label the endothelium has also been widely applied, for example in murine cutaneous squamous cell carcinoma tumors cleared in ECi (41), mammary tumors cleared in fructose (39), melanoma xenografts cleared in BABB, CUBIC and TDE, and primary human cancer biopsies cleared with TDE (59), human colon adenocarcinoma biopsies cleared in FocusClear (14). CD31 labelling was compared to CD34 in a study that used tissue clearing on murine GBM with CUBIC and iDISCO (29), and concluded higher specificity of CD34 antibody for labelling endothelium. CD34 was also used to image the vasculature of mouse and human kidney (44), indicating that it labels endothelium specifically in different tissues. Other markers used in the study of tumor vascularization are VEGFR3, a marker of angiogenesis and CD42c, a platelet marker, used in CUBIC-cleared lung cancer xenografts (36), coagulation factor VIII which was detected in melanoma xenografts (59) and MECA-79, used to visualize high endothelial venules in fibrosarcoma and 4T1 xenografts (60) (**Table 1**).

The lymphatic system, especially tumor draining lymph nodes, is a site of particular interest in cancer research since it is a key hub for cancer cell dissemination and immune education. Lymphangiogenesis and the architecture and function of the lymphatic vasculature plays an important role in maintaining the niche of stem cells in healthy tissues (42, 61) and potentially the niche of cancer stem cells as well. Lymphatics can be labelled using an anti-Lyve1 antibody as demonstrated in the liver (46) and in DCM and DBE cleared bladder cancer resections (43). Lymphatics of PanIN and PDAC lesions were visualized in human and mouse samples with antibodies against Lyve-1 and podoplanin and clearing in RapiClear 1.52 (62). Tissue clearing of lymph nodes has been achieved with techniques such as Ce3D for imaging immune cell populations and vasculature (63–65), and has enabled to track the population dynamics of cells involved in adaptive immunity such as CD8^+^ T cell differentiation (66).

### 2.6 Tissue clearing to image tumor innervation

Cancer innervation is a timely field of research, as depending on the context, nerves can promote or inhibit cancer growth and cancer cells can invade perineurally (67). Neurons can readily be mapped by many clearing protocols, and have been imaged in normal tissues of the human colon (68), pancreas (69), and in a mouse model for pancreatic neoplasia where the GFAP-positive glia and TUJ1-positive neurons were found to be enriched around lesions. This study visualized both innervation and vascularization in the same samples which suggests that angiogenesis and innervation occur in parallel during neoplastic hyperproliferation and early stages of transformation in the pancreas (70).

## 3 IVM: a window to visualize TME-cancer cell interaction dynamics in 4D

Static volume imaging allows the molecular phenotyping in the 3D cancer space but represents a snapshot in time and lacks insight into the dynamics of the same cancer cells over time. The combination of IVM with subsequent 3D molecular analysis by tissue clearing poses an attractive solution combining the strengths of both technologies (71, 72). Recently, IVM has proven extremely useful to shed light on dynamic processes at play in tumor formation and progression, immune escape and metastasis (73–76).

### 3.1 Recent protocols and window optimizations for IVM

**Figure 3:**
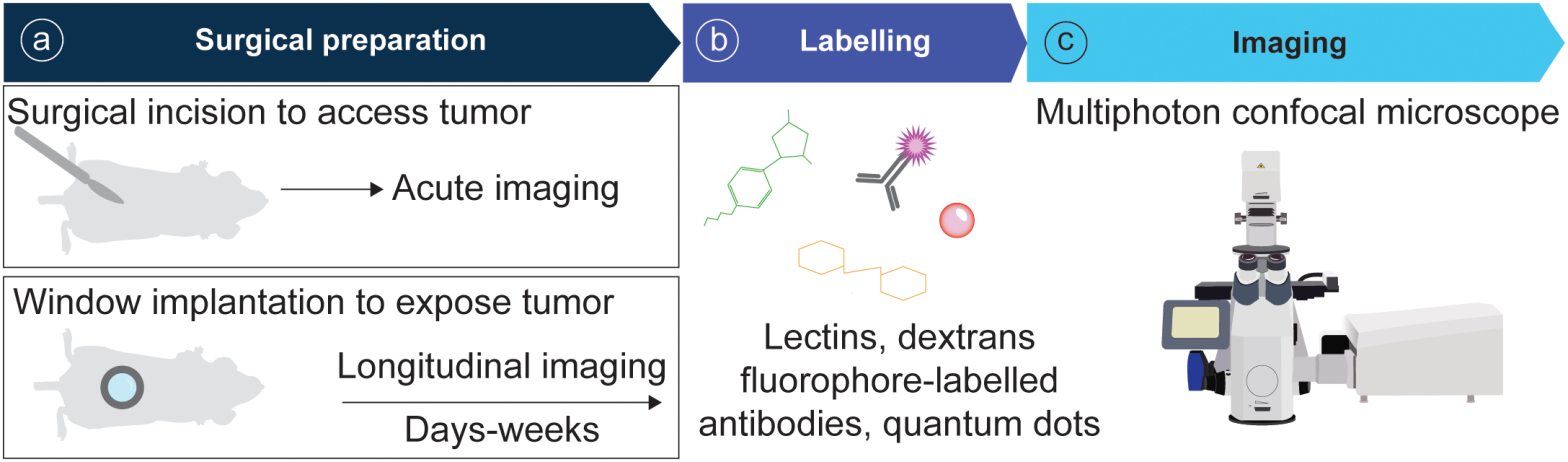
Pipeline of IVM for tumor models. **(A)** Animals are surgically prepared for acute or longitudinal imaging. **(B)** Optionally, mice can be labelled intravenously with fluorescent probes. **(C)** Imaging is performed using a multiphoton confocal microscope.

In recent years the field of intravital microscopy has been broadened and specialized by developments that adapted the technology for different organs. Each organ casts unique demands due to its location that dictates optical accessibility, and due to its organ architecture and underlying physical (and optical) properties. Today an abundance of IVM techniques exists tailored to specific organs. For acute imaging, most organs can be accessed surgically. On the other hand, longitudinal imaging necessitates technologies to grant long-term access to the same organ and region over multiple days to weeks (**Figure 3**). This has for long been accomplished through the help of imaging windows, small sterile rings of solid inorganic material with a microscopy cover-glass inset. Such windows can be surgically implanted in the mouse skin and peritoneum, thereby rendering inner organs optically accessible. In recent years the general window design has been considerably modified to meet unique demands of different implantation sites. Most recent optimizations include windows for the pancreas (77, 78) and thyroid (79), and flexible silicon-based imaging windows for expanding tissues and tumors (80, 81). Several approaches also exist to image cancerous processes in the bone marrow, ranging from the use of endoscopic devices for the femur (82), to the surgical exposure of tibia and calvarium (83, 84).

Some organs, such as those of the respiratory system and the gastrointestinal tract, are under constant movement and need to be stabilized for high resolution image acquisition. For the colon, a window with a stabilizing ferromagnetic scaffold minimizes such motion artifacts specifically during the imaging whilst granting the organ free movement in the imaging breaks to prevent obstruction and maintain intestinal function (85). Using a specialized lung window for longitudinal imaging, Entenberg and colleagues could visualize all stages of the metastatic process, including tumor cell arrival, extravasation, metastatic growth and progression to micrometastases (86).

The dorsal skinfold window represents a widely used alternative to organ specific imaging windows. In this model cancer cells are engrafted subcutaneously in a window chamber implanted in the back skin of the mouse. This approach presents a robust platform to visualize cell-cell interactions in the living organism and may be seen as a straight forward alternative to test hypotheses *in vivo* that were gained in *in vitro* experiments, albeit restricted to questions where the role of the cancer-surrounding normal organ is neglected. Alternative xenograft-based approaches have visualized tumor cell behavior through cancer cell injection in the tongue (87), and engraftment in the eye (88) which allow noninvasive longitudinal IVM without the need of imaging windows.

Once the organ of interest can be optically accessed, different microscopy setups enable to address a multitude of questions. Optical access into deeper tissue layers is hampered by the tissue inherent light scattering and most studies employ 2-photon microscopes to visualize several hundred μm of tissue depth. The recent development of IVM protocols for 3-photon microscopy further increases the imaging depth (89). In addition, fast acquisition speed, for example using a spinning disk or a resonant scanner confocal microscope, can be of advantage for moving organs (90) and allows the capture of highly dynamic events as for example the movement of leukocytes in the blood stream and tumor interstitium (91). The best combination of strategies for optical access and image acquisition will depend on the cancer type and biological question asked. Dedicated protocols for the imaging of primary cancers, metastases and the tumor microenvironment in different sites help identify the optimal strategy (92–95).

Whilst the majority of previous IVM studies focused their attention on cellular behavior, the coming years are poised to see a broadening of IVM utilization with ongoing developments that multiplex bioluminescent and fluorescent reporters and molecular probes (96, 97) to film sub-cellular processes, and approaches that enable the *in vivo* visualization of nanoscale structures by 3D-stimulated emission depletion (3D-STED) microscopy (98), thereby opening the window to view molecular biology in the living organism.

### 3.2 Tools and strategies to visualize cell-cell interactions *in vivo*

Given the ability to access and visualize the anatomic site of interest, a range of fluorescent labels and detection strategies enable the identification of cancer cells and structural and cellular components of the TME in the context of the organ architecture (99). The recent development of a photoactivatable Cre-recombinase mouse allows to target cancerous mutations to specific regions and cells in the organ landscape (100). Cancer cells can be labelled genetically through the expression of fluorescent proteins, and thus traced over sequential imaging sessions of several days to weeks. Proteins that can be activated, or photo-converted to change the fluorescent protein emission spectrum, allow the tracking of individual groups of cells based on their localization (92). With this approach, the colonization of distant organs from breast tumors was mapped by following the dissemination of cancer cells converted in the mammary gland in comparison to cancer cells converted in the lymph node (101).

Proliferation of cancer and TME cells can be visualized indirectly by targeting individual cells with fluorescent proteins for lineage tracing, or directly through genetically encoded fluorescent cell cycle reporters (71, 102). Fluorescent dyes like the recently developed apotracker-red are an option to identify apoptotic cells (103). Today the toolbox of IVM-ready fluorescent labels also includes a range of probes that allow to monitor the activity of the ERK, FAK, TGF-β/SMAD3 and nuclear factor kB (NF-kB) signaling pathways (104–107). A fluorescent reporter of nuclear factor of activated T cell (NFAT) activation was recently used to directly visualize how Treg cells receive TCR signals in the TME (108).

The tumor-surrounding matrix can be readily visualized through second harmonic generations (SHG) detection of fibrillar collagen. Furthermore, recent efforts have targeted individual matrix components with fluorescent proteins. Fluorescently tagged fibronectin (FN) was used to track T cell interaction with FN in the inflamed ear dermis, where Th1 cells were found to migrate along FN fibers (109). The development of a laminin beta1-Dendra2 reporter mouse allows to visualize basement-membrane dynamics *in vivo* and can shed light on how cancer cells invade normal tissues (110). Tumor vasculature is commonly visualized through intravenous injection of fluorescent dextrans, lectins or quantum dots. The choice of dextran molecular weight allows to fine tune this detection to measure vascular permeability (84, 111), and to label phagocytic immune cells (107) (**Figure 3**).

IVM has been instrumental to describe a wide range of fundamental immunological principles and immune cell behaviors (112, 113). Genetic reporters and intravenously injected fluorophore-conjugated antibodies are commonly used to detect immune cells (84, 90). Recent developments for multiplexed *in vivo* antibody labelling through click chemistry offer the opportunity to drastically expand the cell types that can be visualized in the same mouse (114). Nevertheless, it remains challenging to quantify rare cell populations in circulation. Recent developments enable real time imaging of individual fast circulating cells in the blood stream through quantum dots (115), a promising step in the direction of visualizing aggregation dynamics of circulating tumor cells (CTCs), or the interplay between lymphocytes, platelets, and CTCs in circulation *in vivo*.

## 4 Applications of volume imaging to visualize cell-TME interactions in cancer

### 4.1 Cellular relationships between epithelial cells

The crosstalk between cancer cells and their neighboring normal epithelium dictates the biology of an arising lesion already at the earliest stages when only a couple of oncogenic cells start to proliferate. IVM experiments demonstrated how the crosstalk between skin cells bearing oncogenic mutations and their wildtype neighbors can correct aberrant growths (116). In the ear skin, hair follicle stem cells carrying an activating *Hras* mutation outcompeted their wildtype neighbors, yet were integrated into clinically normal skin hair follicles. In contrast, targeting the *Hras* mutation to the upper noncycling region of the skin epithelium led to benign outgrowths (117). Tissue clearing of skin, lung, liver and pancreas showed that the morphological appearance of early lesions is governed by an interplay between the architecture of the host tissue and deregulation of cancer cell actomyosin activity (27, 118). Importantly, the mechanical crosstalk extends to the nuclear level where it causes dynamic changes in nuclear morphology and may threaten genetic integrity (119). Genomic cancer cell evolution and the dynamic TME instruct single cell biology and behavior and lead to diversification in the tumor bulk. IVM demonstrated that this heterogeneity extends to the metabolic level in ER^+^ breast cancer. Using fluorescent biosensors, Kondo and colleagues demonstrated how actin remodeling, phosphatidylinositol 3-kinase (PI3K) and bromodomain activity modulate glycolysis resulting in metabolically distinct tumor cell populations that reside in different areas of a lesion (120). How this regional diversification affects the cellular interactions amongst cancer cells, as well as the interplay of cancer cells with the TME is subject to ongoing investigation. Alongside tumor expansion cancer cells actively remodel their microenvironment, and the TME is understood to be spatially patterned with differing constituents and function in different tumor areas (2).

### 4.2 Interaction of cancer cells and the tumor vasculature

In the study of the TME, vascular remodeling has received particular attention as it impacts tumor nutrition, oxygenation, dissemination, and the recruitment of immune components. Quantitative assessments of the extent of tumor vascularization can be readily achieved by both tissue clearing and IVM, and have shown that several tumor types share a tendency to increase the vascular density within their mass (33, 49, 121). In some cancers the angiogenic capabilities may extend to lymphangiogenesis, as seen in pancreatic cancer (62). The cellular interactions between cancer cells and the tumor vasculature are multifold and reciprocal. IVM experiments on an orthotopic HNSCC model suggest that angiogenesis is specifically induced by a subpopulation of CD44^+^ cancer cells (122). On the other hand, 3D imaging of the tumor-stroma interface in cutaneous SCC showed that the blood vasculature regionally expresses TGF-β, thereby sustaining the cancer stem cell niche and contributing to cancer cell heterogeneity (122).

In an HRAS-driven murine model of cutaneous SCC, angiogenesis was shown through tissue clearing to be triggered during the transition from papilloma to malignant carcinoma. This *de novo* vascularization in late stages of lesion progression facilitates leptin infiltration into the tumor, triggering Pi3k-Akt-mTor signaling, further contributing to the benign to malignant transition (41). Cancer progression through vascular remodeling is also seen in the bone marrow in acute myeloid leukemia (AML), where IVM experiments visualized how the remodeling of endosteal blood vessels abrogates hematopoietic stem cells (HSCs), HSC niches and osteoblasts (123) through physical dislodgment via the damaged vasculature. Pharmacological MMP inhibition reduced vascular permeability and healthy cell loss, thereby limiting AML infiltration, proliferation, and cell migration (124).

### 4.3 Cancer cell crosstalk with the immune microenvironment

The tumor vasculature is a significant route of tumor entry for immune cells that may clear cancer cells or be recruited for their tumor protective behavior. IVM successfully captured the critical steps in the cascade of lymphocyte adhesion to tumor-associated high endothelial venules (TA-HEVs), from initial lymphocyte tethering to the vessel wall to lymphocyte rolling and sticking, and extravasation (125). Tumor-infiltrating lymphocyte (TIL) motility changes both longitudinally during tumor progression (126), as well as regionally. Cytotoxic T lymphocytes (CTLs) show reduced motility and activity in avascular tumor areas but were found to be highly motile in vicinity to peripheral blood vessels and cleared tumor cells around them. Interestingly, blood flow is essential for CTL migration (127). Concomitantly, CTLs were shown to first accumulate in the tumor periphery and subsequently redistribute towards the invasive tumor front (128). Based on their migration patterns, CTLs have been understood to display a tumor cell searching behavior in the tumor periphery, which abates in velocity and changes into a “probing” behavior in tumor cell proximity indicating target engagement (129). IVM imaging showed that in the process of tumor cell killing, multiple CTLs undergo temporary sublethal contacts with individual tumor cells to induce killing in a process of additive cytotoxicity (128). On the other hand, CTL recognition of a few tumor cells can elicit sufficient IFNγ secretion to cover distances of several hundred micrometers and inhibit tumor growth even in regions not frequented by CTLs (130).

Cancers hijack various cellular pathways to evade the immune response. T regulatory (Treg) cells are inherently poised to recognize antigens in the TME, and auto-regulate through short-lived interactions with conventional dendritic cells (cDCs) (108). Neutrophils stimulate T cells in a type I interferon (IFN) signaling dependent manner (131). However, tumor cells can elicit the production of neutrophil extracellular traps (NETs) from neutrophils and granulocytic myeloid-derived suppressor cells (MDSCs), and these NETs enwrap cancer cells, thereby reducing interfaces with CTLs and shielding against cytotoxicity (132). Neutrophils can promote tumor growth by activating MDSC that in turn inhibit the immune response orchestrated by effector T cells against cancer cells, as observed in cleared murine HNSCC tumors (47). Peritumoral neutrophils are more motile than intratumoral neutrophils (133), suggesting a different interactive behavior in specific tumor areas.

TAMs play a multifold role in cancer progression. As for lymphocytes and neutrophils, volume imaging data suggest that TAM behavior differs drastically between tumor subtypes, stage and among different areas of the same lesion. IVM showed that the migratory behavior of TAMs is different in genetically distinct GBMs (134). In an orthotopic model of anaplastic thyroid cancer, tissue clearing suggested that the infiltration of tumor-associated macrophages is significantly higher in the primary tumor than in the metastases, indicating that the microenvironment in which the tumor develops conditions the infiltration of tumor-promoting TAMs (37). Using the dorsal skinfold chamber to track the crosstalk between macrophages and cancer grafts by IVM identified macrophage subpopulations based on their localization in the tumor core or periphery, and found that those populations differed in *Arg1* gene expression and motility. This study also demonstrated the powerful combination of IVM with single cell RNA sequencing to gain molecular insight from a single cell phenotype that can only be observed in the native organ context (135). The cell-cell interaction between TAMs and cancer cells is a driving factor of tumor aggression. Macrophages were found to induce cancer cell stemness though Notch-Jagged signaling (136), and enable cancer cell dissemination through dynamic multicellular interactions (137, 138). In an orthotopic breast cancer model, monocytes were shown to arrive at the tumor as motile streaming TAMs that then differentiated into sessile perivascular macrophages. This differentiation occurred through cancer cell induced TGFβ-dependent upregulation of CXCR4 in monocytes while CXCL12 expressed by perivascular fibroblasts attracted these motile TAMs toward the blood vessels, bringing motile cancer cells with them. Once on the blood vessel, the migratory TAMs differentiated into perivascular macrophages, promoting vascular leakiness and intravasation (137).

Together volume imaging experiments of the recent years demonstrated that tumor- supportive and tumor-inhibiting activities of immune cells crucially depend on the regional microenvironment, and are modulated in a highly dynamic way through complex multi-cellular interactions.

### 4.4 The basement membrane and cancer cell invasion

The basement membrane constitutes a key barrier to tumor cell migration into the surrounding parenchyma. Recent IVM experiments, using a fluorescent laminin beta1 reporter mouse, show that basement membrane components are synthesized by cancer cells as well as endothelial cells in a dynamic and regionally heterogeneous manner. Interestingly, laminin beta1 turnover is higher in cancerous areas compared to normal epithelium, suggesting a local pre-conditioning of the basement membrane preceding tumor cell invasion (110).

IVM imaging of cancer cell invasion has been instrumental to understand the cellular processes and interaction of cancer cell dissemination. Myosin 10 filopodia support the basement membrane in early lesions, but contribute to a proinvasive phenotype later in disease (139). Interestingly, cells that are able to breach the basement membrane, due to experimentally induced loss of E-cadherin which increases cancer cell motility, require further signals in order to disseminate into the surrounding parenchyma (140). Together, 3D cellular confinement, cell density and cell-cell junction stability orchestrate epithelial fluidization and single cell release (141, 142), and set out the paths of migration and leader-follower cell relationships in collective movements.

Cancer cells were found to migrate along collagen fibrils and blood vessels in several tumor models (143, 144). Leader cells undergo significant deformations, potentially due to the confinement by the ECM, but create paths for follower cells that have more normalized cell and nuclear shapes (145). This synchronous migration along common paths may reflect an expansion along newly generated space in the ECM confinement, but could also be instructed directly from leader to follower cells through extracellular vesicle exchange (146, 147). Thereby, the migratory behavior differs throughout the tumor landscape. GBM comprises an invasive margin with slow directed invasion and a diffuse infiltration margin with fast but less directed cell movements. Migration along blood vessels correlated with higher velocity and direction away from the tumor core whereas intraparenchymal migration along white matter tracts was measured to be slower and less directed (148). In glioma, IVM showed that GFAP splice variants fine‐tune invasion by modulating migration persistence. Whilst depletion of either isoform increased the migratory capacity of glioma cells, GFAPδ‐depleted cells migrated randomly through the brain tissue, whereas GFAPα‐depleted cells displayed a directionally persistent invasion into the brain parenchyma (32).

### 4.5 Cancer cell-TME interactions in transit to distant organs

Tumor cell intravasation is a multicellular process in the TME which in breast cancer models was shown to require direct contacts between a proangiogenic perivascular macrophage, a tumor cell overexpressing the actin regulator protein Mena, and an endothelial cell that together create the “tumor microenvironment of metastasis” (TMEM), allowing cancer cell intravasation through transient vascular permeability (137). Vascular travel is a bottle neck in tumor cell dissemination as only a portion of the cells entering the blood stream are able to seed and colonize distant organs. Real-time visualization of circulating tumor cell (CTC) subpopulations through conjugated quantum dots showed that PDAC CTCs expressing the surface protein CD24 have the ability to migrate along vessel walls to distant organs (149). CTC packing in cell clusters can provide support for traveling cancer cells and has been debated to occur through collective intravasation or *de novo* clustering in the circulation. Intravital imaging showed that breast cancer CTC clusters form in circulation through CD44 protein mediated cell aggregation (150).

Tissue clearing showed that breast cancer cells extravasate with the help of actin-rich protrusions (151). CTC extravasation depends on reciprocal interactions between CTCs and endothelial cells of distant organs that can prohibit or permit CTC organ entry. The bone endothelium expresses ephrin-B2 which activates ephrin-B4 in circulating melanoma cells thereby repulsing CTCs from attaching to the bone endothelial cells and barricading against metastasis formation (152). In the mouse lung, extravasation of breast cancer cells is dependent on physical interaction between tumor cells and macrophages in a IL4-CXCR2 dependent manner (153).

### 4.6 Cell-cell crosstalk at metastatic sites

Metastasizing cancer cells can disseminate and establish tumors in organs with disparate cellular and physicochemical characteristics, or can present strong tropism and invade only one secondary organ (154). Tissue clearing of entire mice has shown the diverse tropism of different cancer cell lines such as mammary MDA-231 and pancreatic OS-RC-2 cells, and their colocalization with the vasculature across the mouse body (58). Volume imaging uncovered a widespread heterogeneity in the seeding potential, and the likelihood for successful metastatic outgrowth of different tumor cell subpopulations. These factors are now understood to be modulated by cell intrinsic properties and CSC traits on the one hand, as well as by dynamic multi-cellular contacts at the metastatic site on the other hand.

Multicellular interactions influence the survival and outgrowth of metastasis seeding cancer cells. In the brain, early extravasating cells may be cleared by phagocytosis from TAM and microglia, although these effects abated in later stages of metastasis formation (155). As in primary tumors, the multi-cellular arrangements of cancer cells and TME components in metastatic sites show spatial and longitudinal heterogeneity. There is a highly heterogeneous cellular interplay between metastases even from the same tumor, as illustrated in breast cancer brain metastases that involve glial cells and astrocytes of the brain parenchyma to very different extents (33).

The communication between cancer cells and the TME at different organs may amplify the heterogeneity seen in metastatic lesions. Indeed, the proliferation dynamics of cancer cells were found to depend on the organ colonized as shown with A549 lung adenocarcinoma cells after intracardiac administration into mice (156). One environmental mechanism may involve the access of pro-and antitumorigenic immune cells. Neutrophils are highly motile in lung capillaries and change their behavior to slow patrolling and stationary upon recruitment to lung metastasis (90) where they were filmed to correlate with tumor cell proliferation (157).

## 5 Volume imaging as a platform for therapy development

Whilst historically tissue culture assays have been used to carry out cancer drug screens, an ever-growing body of literature highlights limitations in the predictive value of cell culture assays to extrapolate the effect of drugs from *in vitro* to an entire organism. This may be attributed to a difference in signals that cells cultured *in vitro* lack in comparison to cancer cells *in vivo*. Cancer cells receive stimuli from neighboring cancer cells, non-tumor cells, architectural constraints and systemic physical factors that shape tumor biology *in vivo*. Recent studies using IVM showed that even cell-intrinsic molecular characteristics may not be adequately reflected in 2D/3D culture systems (158, 159). In fact, when transplanted *in vivo*, a panel of breast cancer cell lines showed remarkable cellular heterogeneity, even in the same nodule that was not observed *in vitro (* 160). Hence, preclinical models remain essential to evaluate the effect of new therapeutic agents (161). Combining preclinical models with fluorescence volume imaging constitutes a powerful tool to assess potential anti-cancer treatments, many of which directly targeted against components of the TME, and to study how they affect the interaction between cancer cells and the TME.

### 5.1 Drug delivery is dependent on tumor vascularization and perfusion

Volume imaging technologies pose an attractive platform to evaluate a new drug, map off-target effects and identify the mechanisms underlying acquired therapy resistance.

Several studies used tissue clearing to assess the therapeutic delivery of drugs and drug vehicles *in vivo*. The distribution of nanoparticles and PEGylated liposomes, and their spatial relation with the vasculature was used to evaluate efficiency of drug delivery into the tumor (56, 162). Similarly, tissue clearing visualized tumor perfusion by fluorescently tagged anti-tumor antibodies, such as the HER-2-targetting antibody trastuzumab, and a fluorescent analogue of the chemotherapeutic agent docetaxel (35, 55). In cases where the therapeutic agent cannot be traced directly, drug interference with fluorescent signals from the target cells may serve as proxy for measuring drug engagement. In an elegant example, doxorubicin uptake was measured *in vivo* on a single cell level by exploiting the changes in fluorescence lifetime of histone-GFP fusions upon doxorubicin binding to chromatin. This uncovered drastic differences in doxorubicin binding between different peritoneal metastases in the same mouse, as well as different regions and cells in the same lesion (163).

In a murine model of non-Hodgkin lymphoma, tissue clearing revealed that the delivery of therapeutic CD20 fragment antigen-binding (FAB) antibody regions was dependent on tumor perfusion (164). IVM showed that preserving endosteal vessels in AML improves the efficacy of chemotherapy (123). IVM of melanoma and PDAC xenografts implanted under a dorsal skinfold window was used as a platform to quantify the vascular effects of acute cyclic hypoxia and antiangiogenic treatment (165).

A key goal of treatment development has been to educate and exploit the immune system to reject cancer progression. Immune infiltration into the tumor is also dependent on its vascularization. IVM assays are an elegant tool to measure the biodistribution and efficacy of CAR T cells, as demonstrated for primary central nervous system lymphoma (PCNSL) and GBM (166, 167). Volume imaging identified tumor associated high endothelial venules (TA-HEVs) as the main sites of lymphocyte arrest and extravasation into tumors that received anti-PD-1/anti-CTLA-4 immunotherapy. Increasing TA-HEV endothelial cells (TA-HEC) frequency and maturation improved CTL recruitment and therapy response (125). IVM experiments showed that carboplatin chemotherapy profoundly alters the TME to promote lymphocyte adhesive interactions with the tumor vasculature resulting in improveed lymphocyte trafficking (168). Another IVM study in a GBM model suggested that radiotherapy may aid CAR T cell extravasation and expansion (167). CTLs engineered to overexpress the chemokine receptor CXCR4 displayed increased homing to the bone marrow and engraftment, and acquired greater anti-tumor immunity (169). Cancer-derived adenosine weakens the physical interaction between activated effector CTLs and target cells, providing cancer cells with an escape way from immunotherapy. IVM showed that antagonization of adenosine A2a receptor (ADORA2a) signaling improved CTL-target engagement and cancer cell killing (170).

### 5.2 Measuring drug efficacy

A key aspect of therapy development is the measure of *in vivo* drug efficacy. IVM of calvarium bone marrow tracked the migratory behavior of acute myeloid leukemia (AML) cells in comparison to T cell acute lymphoblastic leukemia (T-ALL), and demonstrated distinct traits of the lineages in their response to chemotherapy and CXCR4 antagonism, highlighting the importance of carefully evaluating cancer traits *in vivo* for each tumor type (171). Tissue clearing has been beneficial to evaluate therapy efficiency in various preclinical cancer models, such as HepG2 xenografts to assess the severing of the vasculature induced by antiangiogenic therapeutic siRNAs (52), and HER-2-expressing breast cancer xenografts treated with colony-stimulation factor 1 receptor inhibitor PLX3397, which reduced tumor burden without impairing the infiltration of macrophages (35). Tissue clearing for whole organ quantifications of metastatic burden showed that targeting macrophages with clodronate liposomes reduced lung colonization by intravenously injected A549 cancer cells (36).

KPL-4 breast cancer xenografts were found to present a normalized vasculature when treating mice with anti-VEGF antiangiogenic therapy (172), indicating an improvement of tumor irrigation and of the penetration of nutrients and oxygen, but potentially also of other therapeutic agents that may hence more efficiently find and eliminate cancer cells.

### 5.3 Immunotherapy

Immunotherapy is efficient in immunogenic tumors that express immune checkpoint molecules, whether in the cancer cells or in other infiltrating cells of the TME. Tissue clearing has been used to map the expression of immune checkpoint molecule PD-L1 and immune cells throughout tumors such as MMTV-HER2/Neu (39, 173) and NSCLC patient resections (174). Such 3D quantifications are more precise than 2D analyses of tissue sections and could be applied to patient biopsies as a prognostic marker that predicts the response to immunotherapy. In addition to tissue clearing, IVM has been successfully applied to evaluate the biodistribution of immunotherapies (175). Furthermore, such *in vivo* assays have proven useful to pinpoint the reasons for immunotherapy failure, and could demonstrate for example how a fluorophore-conjugated PD-1 targeting antibody shown to target PD-1^+^ tumor infiltrating CD8 T-cells was immediately cleared by PD-1^-^ tumor associated macrophages, preventing tumor regression (176).

### 5.4 Cancer therapy side effects

One key advantage of tissue clearing is the power to detect rare events in large tissue volumes in a quantitative manner and at little time expenditure. These traits make tissue clearing an attractive solution to study side effects of cancer therapies. Once organ sites suffering under off-target effects and drug toxicity are identified, IVM can add to this informative value by revealing the dynamic interplay of cell populations. Liver toxicity is a common side effect of viral therapies and Naumenko and colleagues demonstrated how IVM can be used to track the interactions between hepatocytes, Kupffer cells, CD8 T-cells and neutrophils to study oncolytic adenovirus associated liver toxicity (177). Apart from insights that educate drug refinement, IVM may also uncover new treatment options. For example, IVM experiments uncovered evidence that bisphosphonate drugs, which were designed to target bone, readily accumulate in microcalcifications in breast cancers where they have been suggested to stimulate TAMs and improve cancer patient survival independent of the drug’s antiresorptive effects on bone tissue (178).

### 5.5 Therapy resistance

A common trait of many cancers is the ability to acquire therapy resistance over the course of a drug treatment. The underlying mechanisms can be partially attributed to genomic and epigenomic evolution and molecular adaptation of tumor cell subpopulations. The process of epithelial-to-mesenchymal transition (EMT) has been under intense debate to play a key role in cancer aggression and IVM experiments proposed that cancer cells that have undergone EMT may be a source for cancer recurrence upon chemotherapy (179). Volume imaging suggests that the acquisition of therapy resistance, coming from subpopulations in the tumor bulk, may be linked to specific architectural regions of a tumor lesion. Indeed, cells in the invasive front were found to display a higher degree of radiotherapy resistance and survival. Combinatorial integrin targeting sensitized these cells and abrogated tumor relapse (180). Apart from tumor cell intrinsic molecular paths towards drug resistance, IVM experiments also demonstrated the role of direct interactions and crosstalk between tumor cells and the TME in this context. Matrix stiffness was found to dictate PDAC chemosensitivity and IVM showed that therapy priming through focal adhesion kinase (FAK) inhibition pulses could drastically increase PDAC cell sensitivity to gemcitabine/abraxane (104).

## 6 Future perspectives in volume imaging of the TME

Since its rediscovery about a decade ago, tissue clearing has been drastically refined to meet the most diverse problems in cancer research, from the study of cancer cell interactions with the infiltrating TME to the mapping of tumor vascularization and innervation, and quantifications of whole-body metastatic load. A wealth of excellent techniques exists tailored to different organs, cancers, and fluorescent readouts. The recent development of accessible whole mouse clearing techniques and antibody labelling of cells spread throughout the entire organism (181) allows to study the differential TMEs at both the primary tumor and metastatic sites in the same organism.

Today most of the tissues and architectural structures can be visualized in 3D. Open opportunities remain in the detection of pathogens, such as whole organ quantifications of the distribution of microbiota in tumors (182).

One bottleneck of tissue clearing remains in the limited detection of multiple cell types in the same sample. However, technical solutions may not be far as pioneering studies on 2D tissue sections have pushed the boundaries in multiplexing for fluorescent microscopy. Imaging mass cytometry detects up to 32 markers (183), and 3D mass cytometry has recently been achieved for thick sections of around 300 um depth (184).

Whilst tissue clearing constitutes a powerful tool to examine entire tissues, organs and organisms, refractive index matching can be combined with expansion microscopy to visualize subcellular details (185). With this technology, super-resolution information of the molecular cancer cell-TME interactions can be unveiled.

In order to connect 3D information with an understanding of cellular dynamics, IVM today offers several platforms, such as the dorsal skin fold chamber, tumor grafts in the eye or tissue-engineered bone constructs with a skin window for longitudinal imaging. Together they enable cancer research to evaluate potential therapeutics in a more relevant setting *in vivo* without the expenditure of developing strategies to study cancers in their native organ site (88, 176, 186). Tumor cell grafts readily remodel their surrounding vasculature and stand in intense crosstalk with the immune system, providing ample opportunity to understand and interfere with fundamental cancer traits such as tumor angiogenesis and immune evasion. However, these strategies may give little opportunity to study the crosstalk between cancer cells and their neighboring normal epithelial cells. Furthermore, the tumor graft origin from *in vitro* cultures may fundamentally alter cancer cell properties. Indeed, 3D imaging showed that the angiogenic capacity of xenograft models depends on the culture conditions prior to injection, suggesting that epigenetic changes *in vitro* condition the communication between cancer cells and the vascular microenvironment *in vivo *(50). Furthermore, intravital imaging of lung metastases showed that spontaneously disseminating tumor cells are more capable to form distant metastasis than experimentally metastasized tumor cells (187).

Whilst the majority of volume imaging studies have used these platforms for fundamental and preclinical research, 3D imaging also poses considerable attractiveness for cancer staging. Tissue clearing could be exploited for the analysis of cancer patient resections in a diagnostic setting. 3D mapping of vasculature and lymphatics was shown to improve the prognostic evaluation of bladder cancer (44, 60). The presence of high endothelial venules in models of fibrosarcoma and mammary carcinoma was found to correlate with tumor regression upon Treg cell depletion (46), and to predict survival and αPD-1/αCTLA-4 immunotherapy response of melanoma patients (126). Pathways for 3D image automation and standardization are already being laid out, as demonstrated in the successful computational segmentation of prostate cancer patient samples (22). A 3D high-resolution view into a clinical cancer resection holds great informational value and can uncover unexpected subtleties in the cellular composition and spatial arrangements (2). For example, tissue clearing showed that luminal prostate cancer, widely expressing K8 (marker of luminal epithelium) contains regions enriched in K5^+^ myoepithelium which acquire a more differentiated, ductal conformation (23). Nevertheless, clinical adoption and utilization will require careful evaluation of 3D imaging data and side by side comparisons with conventional histopathological assessment to educate data interpretation towards the best treatment decisions and to avoid patient overtreatment.

## Author contributions

JA and HM researched literature for the review, discussed content, wrote, reviewed and edited the manuscript. All authors contributed to the article and approved the submitted version.
